# Telehealth vs Clinic Postoperative Visit After Hysterectomy: A Randomized Controlled Trial

**DOI:** 10.1007/s00192-025-06070-9

**Published:** 2025-01-31

**Authors:** Susan D. Wherley, David Sheyn, Leah H. Hellerstein, Hope Bauer, Jeffrey Mangel, Sarah Sears, Linda-Dalal Shiber, Robert Pollard

**Affiliations:** 1 Urogynecology and Reconstructive Pelvic Surgery, University Hospitals Cleveland Medical Center, Cleveland, OH USA; 2 Department of Obstetrics and Gynecology, MetroHealth Medical Center, Cleveland, OH USA; 3 Urology Institute, University Hospitals Cleveland Medical Center, Cleveland, OH USA

**Keywords:** Hysterectomy, Postoperative care, Patient satisfaction, Telehealth, Telemedicine

## Abstract

**Introduction and Hypothesis:**

Telehealth is becoming more common, but there is a paucity of literature investigating the role of telehealth in perioperative gynecologic care. The authors hypothesized that patients evaluated via telehealth 4 weeks after minimally invasive hysterectomy would not have lower satisfaction than patients evaluated in clinic.

**Methods:**

This was a randomized controlled noninferiority trial of patients who underwent minimally invasive hysterectomy at a single academic medical center. Participants were randomized to postoperative clinic visit or telehealth visit 4 weeks after hysterectomy. After the 4-week postoperative visit, patients were sent a satisfaction questionnaire. The primary outcome was overall patient satisfaction on a 100 mm visual analog scale. Secondary outcomes were 90-day postoperative complications and unplanned events.

**Results:**

One hundred one patients who underwent minimally invasive hysterectomy were identified for inclusion. Complete data were collected for 47 in the clinic group and 45 in the telehealth group. Overall postoperative visit satisfaction did not differ between groups (94.3 clinic vs. 92.0 telehealth, *p* = 0.47). The clinic group was significantly more likely to contact the clinic two or more times (*p* = 0.02); both groups were similarly likely to contact the clinic at least once (57.4% vs. 51.1%). Postoperative complications did not differ between groups, nor did unplanned clinic visits or emergency department (ED) visits.

**Conclusions:**

Postoperative visit satisfaction of patients evaluated via telehealth was noninferior to the satisfaction of patients seen in the clinic 4 weeks after minimally invasive hysterectomy. Unplanned clinic visits and ED visits did not differ between groups, nor did 90-day postoperative complications.

**Supplementary Information:**

The online version contains supplementary material available at 10.1007/s00192-025-06070-9.

## Introduction

Telehealth, or the provision of medical care at a distance using technology, is becoming increasingly common, partly due to the SARS-CoV-2 pandemic. Known benefits of telehealth include improved access to care, convenience for patients, and savings of time and cost without sacrificing quality of care or patient satisfaction [1, 2].

The use of telehealth for postoperative care is well-described in the general surgery literature, with more than 10 years of data reporting similar rates of detection of postoperative complications, follow-up, and satisfaction for procedures, including hernia repair, laparoscopic cholecystectomy, and laparoscopic appendectomy [3–7]. However, there is a paucity of literature investigating the role of telehealth in perioperative care of gynecologic patients. There is only one small study investigating postoperative telehealth visits after laparoscopic hysterectomy or excision of endometriosis. That study of 41 patients found significantly higher patient satisfaction with telehealth visits than clinic visits [8].

Postoperative visits include discussion of the patient’s postoperative course, pathologic results, and any questions or concerns. Given the low rates of postoperative complications after minimally invasive hysterectomy or diagnosis of occult cancer on pathologic examination, these visits are frequently brief [9, 10]. Patients often spend significantly more time and effort transporting to the clinic and waiting to be seen than with their surgeon.

The aim of this study, therefore, was to assess whether patient satisfaction, postoperative complications, and unplanned visits differ between postoperative hysterectomy patients evaluated via telehealth and in clinic. The authors hypothesized that postoperative patients evaluated via telehealth 4 weeks after minimally invasive hysterectomy would not have lower satisfaction than patients evaluated in clinic.

## Materials and Methods

This was a randomized controlled noninferiority trial of patients who underwent minimally invasive hysterectomy for non-oncologic indications between August 10, 2022, and July 12, 2023, at a single academic medical center. This study was approved by the Institutional Review Board at our institution. English-speaking adult patients planning to undergo minimally invasive hysterectomy for non-oncologic indications were identified for inclusion at their preoperative visits. Exclusion criteria included lack of reliable access to a smartphone, inability to complete an online questionnaire in English, intraoperative injury, blood transfusion, postoperative stay greater than one day, and diagnosis of occult cancer on pathology.

Participants consented at their preoperative visit prior to scheduled minimally invasive hysterectomy. After discharge from the hospital following the minimally invasive hysterectomy, patients were randomized in a 1:1 ratio into two study groups: telehealth visit or clinic visit. Randomization occurred after discharge from the hospital to minimize post-randomization dropout owing to intraoperative or immediate postoperative exclusion criteria. Randomization was performed using blocking with a block size of 10 and a 1:1 allocation ratio. The randomization sequence was generated using a random number table and placed into sealed envelopes to be opened at the time of discharge by the appointment scheduling team. Participants were then called by the scheduling team to arrange a postoperative visit 4 weeks after surgery either in clinic or via telehealth. A single postoperative visit 4 weeks after surgery is the long-standing standard of care at our institution.

Care between the time of discharge and the scheduled postoperative visit 4 weeks after surgery was the same for both groups. Patients were encouraged to call the clinic with any concerning symptoms or questions about their surgery. After the 4-week postoperative visit with their surgeon, patients were sent an email link for a questionnaire through REDCap (Research Electronic Data Capture), a secure, web-based software platform designed to support data capture for research studies [11, 12]. The study questionnaire, available as Supplement 1, included seven or ten questions about postoperative visit satisfaction, depending on whether the patient had a clinic or telehealth visit, as well as three questions about transportation to clinic and prior telehealth experiences. The study questions were derived from a California Health Care Foundation document on building surveys to measure patient experience and satisfaction with telemedicine [13]. A conscious decision was made by the study team to utilize these questions tailored to postoperative care, rather than a validated satisfaction questionnaire such as the patient satisfaction questionnaire short form (PSQ-18), which assesses patient experiences with the healthcare system at large, or the surgical satisfaction questionnaire (SSQ-8), which assesses patient perceptions of their surgical procedure and immediate recovery period [14, 15]. Neither the PSQ-18 nor the SSQ-8 have any questions that address postoperative physician encounters and therefore are inadequate for assessing a patient’s experience or satisfaction with a postoperative visit. If participants did not complete the REDCap questionnaire within 2 days, a reminder email was sent. If they did not complete the questionnaire within 1 week, they were called by a study investigator and reminded to complete the questionnaire. Demographics were collected via the Epic electronic medical record (EMR), including patient age, race, BMI, insurance type, day of discharge, prior surgeries, and concomitant surgeries.

The primary outcome was overall patient satisfaction on a 100 mm visual analog scale (VAS). Secondary outcomes were 90-day postoperative complications and unplanned events, including clinic calls or MyChart messages, clinic visits, emergency department visits, and hospital admissions.

An a priori power analysis determined that if there was truly no difference between the standard and experimental treatment (difference in VAS less than or equal to 15 mm with a maximum standard deviation of 30 mm per patient), then 82 patients would be required to be 80% sure that the upper limit of a one-sided 95% confidence interval would exclude a difference in favor of the standard group of more than 5%. A loss rate of up to 20% was anticipated; therefore, we planned to randomize 50 participants per group for a total study population of 100 patients. Groups were stratified into telehealth and standard clinic follow-up arms. Pairwise analysis was performed using Student’s *t*-test, Wilcoxon rank-sum and Fisher’s exact test as appropriate. Descriptive statistics are expressed as means with standard deviations. Statistical analysis was performed using STATA version 14.1 (Stata Corp, College Station, USA).

## Results

**Fig. 1:**
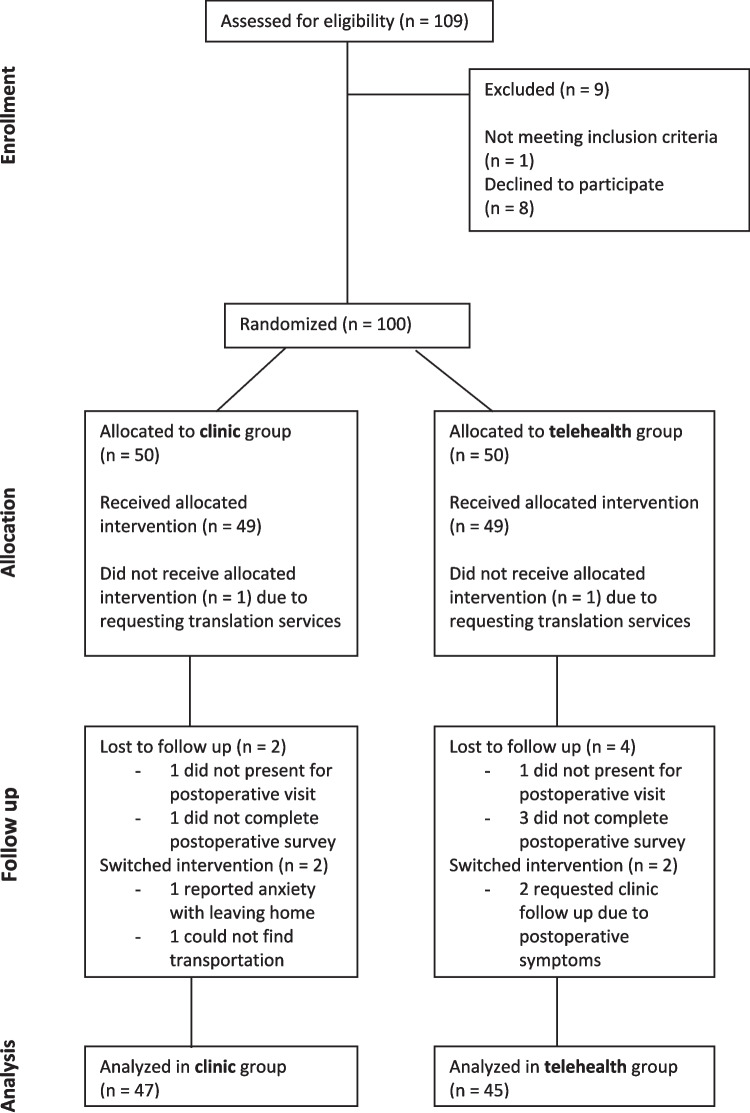
CONSORT diagram of patient participation

Figure 1 shows a flow chart of patient recruitment and participation. One hundred one patients who underwent minimally invasive hysterectomy were identified for inclusion. One patient consented to participation, but was not randomized due to a longer than anticipated inpatient admission for a chest hematoma related to a concomitant procedure. Two patients were disqualified after randomization after clarifying that they were less comfortable speaking English than originally understood by the research team. Two patients did not present for a postoperative appointment at any point, even after several attempts to reschedule. Four patients did not complete the survey after their postoperative appointment. Complete data was collected for 92 patients, 47 in the clinic group and 45 in the telehealth group. Four patients switched groups prior to their scheduled postoperative visit; two patients from the clinic group to the telehealth group and two patients from the telehealth group to the clinic group. One patient who switched from the clinic group to the telehealth group cited anxiety when leaving the house secondary to post-traumatic stress disorder and the other cited an inability to obtain a ride to the clinic. Both patients who switched from the telehealth group to the clinic group cited clinical concerns that they wanted to discuss with their surgeon in person. All analysis was performed by the intention-to-treat principle.

Table 1 displays patient demographics. Demographics were not significantly different between groups, with the exception that more patients in the clinic group had a BMI ≥ 40. The average age was 43.1 years, ranging from 21–81 years. One quarter of patients were aged 50 or older. One third of the study population was Black and nearly 10% were of Hispanic ethnicity. Half of the patients had private insurance and the remaining half had public insurance or were uninsured. The majority of patients underwent total laparoscopic hysterectomy (88%); the rest underwent total vaginal hysterectomy (12%). Concomitant procedures are listed in Table 1.

**Table 1:** Patient characteristics, reported as *n* (%) unless marked with asterisk*, indicating mean (SD)

	Clinic (*n* = 47)	Telehealth (*n* = 45)	*p* value
Age*	44.1 (11.8)	42.0 (12.0)	0.39
Race			0.42
White	33 (70.2%)	28 (62.2%)	
Black	14 (29.8%)	17 (37.8%)	
Hispanic ethnicity	2 (4.3%)	6 (13.3%)	0.12
Insurance type			0.56
Medicaid	19 (40.4%)	16 (35.6%)	
Medicare	2 (4.3%)	3 (6.7%)	
Private	25 (53.2%)	21 (46.7%)	
Uninsured	1 (2.1%)	5 (11.1%)	
Body mass index			
< 18.5	1 (2.1%)	1 (2.2%)	0.97
18.5–24.9	8 (17.0%)	10 (22.2%)	0.53
25–29.9	8 (17.0%)	9 (20.0%)	0.71
30–34.9	10 (21.3%)	11 (24.4%)	0.39
35–39.9	8 (17.0%)	11 (24.4%)	0.38
≥ 40	12 (25.5%)	3 (6.7%)	0.01
Prior surgeries			
Lap excision of endometriosis	8 (17.0%)	7 (15.6%)	0.85
Lap adnexal surgery	10 (21.3%)	13 (28.9%)	0.41
1 Cesarean delivery	5 (10.6%)	5 (11.1%)	0.94
2 Cesarean deliveries	3 (6.4%)	2 (4.4%)	0.68
3 Cesarean deliveries	5 (10.6%)	1 (2.2%)	0.58
4+ Cesarean deliveries	2 (4.3%)	1 (2.2%)	0.31
Open adnexal surgery	9 (19.1%)	7 (15.6%)	0.65
Open myomectomy	1 (2.1%)	2 (4.4%)	0.54
Bariatric surgery	2 (4.3%)	3 (6.7%)	0.61
Cholecystectomy	6 (12.8%)	6 (13.3%)	0.93
Appendectomy	9 (19.1%)	6 (13.3%)	0.46
Hernia repair	3 (6.4%)	6 (13.3%)	0.27
Hysterectomy type			0.13
Total laparoscopic	39 (83.0%)	42 (93.3%)	
Total vaginal	8 (17.0%)	3 (6.7%)	
Concomitant procedures			
Bilateral salpingectomy	30 (63.8%)	29 (64.4%)	0.95
Bilateral salpingo-oophorectomy	14 (29.8%)	12 (26.7%)	0.74
Cystoscopy	47 (100.0%)	45 (100.0%)	
Uterosacral ligament suspension	11 (23.4%)	13 (28.9%)	0.39
Midurethral sling placement	7 (14.9%)	4 (8.9%)	0.38
Excision of endometriosis	15 (31.9%)	13 (28.9%)	0.76
Ureterolysis	14 (29.8%)	10 (22.2%)	0.41
Enterolysis	12 (25.5%)	11 (24.4%)	0.91
Joint procedure	2 (4.3%)	3 (6.7%)	0.61
Mini laparotomy	5 (10.6%)	3 (6.7%)	0.51
Discharge day			0.48
POD#0	28 (59.6%)	30 (66.6%)	
POD#1	19 (40.4%)	15 (33.3%)	

Table 2 shows the results of the postoperative satisfaction survey. Overall postoperative visit satisfaction, as determined by response to the prompt “Overall, I was satisfied with my visit today,” on a 100 mm VAS did not differ between groups (94.3 clinic vs. 92.0 telehealth, *p* = 0.47). There were no significant differences between groups for any of the satisfaction questions on the postoperative visit survey, including experience with surgeon attentiveness, questions answered, and time spent with the surgeon. Patients in the telehealth group reported high satisfaction with the convenience of the visit and high likelihood of using telehealth again and recommending it to others. Patients in neither group expressed a preference for the opposite type of visit; this question was associated with the highest standard deviation in both groups.

**Table 2:** Patient postoperative visit survey results, reported as mean (SD) of VAS 100 mm scores

	Clinic (*n* = 47)	Telehealth (*n* = 45)	*p* value
My surgeon listened carefully to me	93.6 (12.1)	94.3 (12.1)	0.78
My surgeon explained the surgical findings in a way that was easy to understand	93.3 (11.4)	93.1 (15.0)	0.95
My surgeon spent enough time with me	93.7 (14.1)	92.9 (14.6)	0.79
My questions regarding my surgery and recovery were answered	95.0 (10.6)	95.1 (10.2)	0.95
I achieved my treatment goals today	93.3 (13.5)	90.7 (14.8)	0.33
Overall, I was satisfied with my visit today	94.3 (11.9)	92.0 (18.9)	0.47
Clinic group only			
I would have preferred to have my postoperative appointment via telehealth	38.4 (33.3)		
Telehealth group only			
I would have preferred to have my postoperative appointment in person Telehealth made it convenient for me to have an appointment with my surgeon I would use telehealth again I would recommend telehealth to someone in my position		33.9 (30.2) 96.0 (10.9) 89.4 (21.2) 87.1 (22.7)	

Table 3 lists postoperative complications and unplanned events. The clinic group was significantly more likely to contact the clinic two or more times through calls or MyChart messages (*p* = 0.02), though both groups were similarly likely to contact the clinic at least once (57.4% vs. 51.1%). Common reasons for contacting the clinic included questions about medical leave-related paperwork, when and how to take medications, and when to remove dressings. Patients also called to report concerns such as pelvic or bladder pressure, incision drainage, elevated temperature, and gastrointestinal symptoms, including constipation, diarrhea, nausea, and bloating. Most questions and concerns were able to be addressed over the phone or through EMR messaging and few led to additional clinic visits. Postoperative complications were rare and did not differ between groups, nor did unplanned clinic visits or emergency department visits. All postoperative complications were Clavien-Dindo grade 1 or 2 [16]. No hospital readmissions occurred.

**Table 3:** Postoperative complications and unplanned events, reported as *n* (%)

	Clinic (*n* = 47)	Telehealth (*n* = 45)	*p* value
90-day complications			
None Urinary tract infection Superficial thrombophlebitis	46 (97.9%) 1 (2.3%) 0 (0.0%)	43 (95.6%) 1 (2.2%) 1 (2.2%)	0.89 0.98 0.31
Clinic calls or messages			
0 Calls 1 Call 2+ Calls	20 (42.5%) 9 (19.1%) 18 (38.3%)	22 (48.9%) 16 (35.6%) 7 (15.6%)	0.54 0.08 0.02
Unscheduled clinic visits			
0 Visits 1 Visit 2+ Visits	39 (83.0%) 7 (14.9%) 1 (2.1%)	41 (91.1%) 4 (8.9%) 0 (0.0%)	0.25 0.38 0.33
Emergency department visits			
0 Visits 1 Visit 2+ Visits	41 (87.2%) 5 (10.6%) 1 (2.1%)	42 (93.3%) 2 (4.4%) 1 (2.2%)	0.33 0.27 0.97

Finally, Table 4 demonstrates patient self-reported experiences with transportation to clinic and prior telehealth appointments. The majority of patients in this study lived within 15 min of their clinic site and used their own vehicle for transportation to appointments. Most had experienced at least one telehealth appointment prior to participation in this study and almost a quarter reported five or more prior telehealth appointments.

**Table 4:** Self-reported patient experience with clinic transportation and telehealth, reported as *n* (%)

	Clinic (*n* = 47)	Telehealth (*n* = 45)	*p* value
Travel time to clinic (round trip)			
< 15 min 15–30 min 30–60 min > 60 min	9 (19.1%) 25 (53.2%) 10 (21.3%) 3 (6.4%)	10 (22.2%) 15 (33.3%) 14 (31.1%) 6 (13.3%)	0.72 0.06 0.28 0.26
Transportation method			
Own vehicle Borrow vehicle/get a ride Taxi/rideshare Public transportation	40 (85.1%) 5 (10.6%) 2 (4.3%) 0 (0.0%)	38 (84.4%) 4 (8.9%) 1 (2.2%) 2 (4.4%)	0.93 0.78 0.58 0.15
Prior telehealth visits			
None < 5 5–10 11–15 > 15	25 (53.2%) 15 (31.9%) 2 (4.3%) 2 (4.3%) 3 (6.4%)	14 (31.1%) 15 (33.3%) 9 (20.0%) 2 (4.4%) 4 (8.9%)	0.03 0.88 0.02 0.96 0.65

## Discussion

In this randomized controlled trial, postoperative visit satisfaction of patients evaluated via telehealth was noninferior to the satisfaction of patients seen in the clinic 4 weeks after minimally invasive hysterectomy. Unplanned clinic visits and ED visits did not differ between groups, nor did 90-day postoperative complications.

Historically, the pelvic exam was considered to be an essential component of the postoperative visit, in theory to detect asymptomatic vaginal cuff dehiscence. However, Caskey et al. demonstrated in a retrospective cohort that vaginal cuff exam after laparoscopic hysterectomy was neither predictive of nor protective against the risk of future vaginal cuff dehiscence, obviating this indication for a postoperative pelvic exam [17]. Some surgeons performing prolapse repair at the time of hysterectomy may still choose to do a postoperative pelvic exam to establish a new baseline POP-Q in case a patient returns with recurrent prolapse concerns in the future. However, while this information may provide a data point for the surgeon analyzing the outcome of their repair, even rapidly recurrent prolapse does not prompt any action in the immediate postoperative period. Given that approximately 30% of patients may eventually require future surgery for recurrent prolapse, which should be determined by patient symptoms rather than by physician assessment of POP-Q, subjecting patients to a postoperative pelvic exam is a futile endeavor by surgeons [18].

This debunking of the necessity of the postoperative pelvic exam raised questions about the necessity of an in-person postoperative visit, which prompted the development of this trial. In our investigation, we found no significant differences in overall patient satisfaction between the telehealth and clinic groups. Patients in both groups reported high levels of satisfaction with their postoperative visits, which aligns with previous research indicating comparable levels of satisfaction between telehealth and in-person care across various medical specialties. These findings suggest that telehealth may offer a satisfactory alternative for postoperative care after minimally invasive hysterectomy, providing patients with greater convenience without compromising satisfaction.

Furthermore, our study revealed no difference in postoperative complication rates and no clinically significant differences in unplanned events. The low rates of postoperative complications observed in both groups underscore the rarity of adverse outcomes after minimally invasive hysterectomy and the safety and effectiveness of telehealth for monitoring patients when counseled appropriately. Interestingly, the majority of patients had at least one unplanned encounter after hysterectomy, regardless of their type of postoperative visit. This is consistent with prior findings from Lua-Mailland et al., who noted that 75% of patients had at least one unplanned encounter within 6 weeks of apical prolapse repair [19]. The vast majority of unplanned encounters in our study were patient calls or EMR messages. While all patients are provided with comprehensive discharge instructions, which include recommended medication schedules, information about wound care, common benign symptoms after hysterectomy, and reasons to call the clinic or report to the emergency department, we found that most calls and EMR messages were related to information covered in the discharge instructions. This may indicate a lack of clarity, a lack of patient comprehension, or distrust in the information provided. Further research is needed to elucidate whether an improved method of conveying postoperative information to patients might reduce healthcare utilization. Additionally, further guidance on patient symptoms necessitating in-person evaluation would help standardize postoperative care when implementing a telehealth-based care plan.

Of note, the largest standard deviations in the satisfaction survey were observed in response to whether patients would have preferred the opposite type of visit than the one they were assigned. Patients in both groups were nearly equally unlikely to prefer the other type of appointment, demonstrating the general acceptability of both types of postoperative visit, but the large standard deviations indicate that some patients in each group would have preferred the opposite visit type. This highlights the important point that patients may have a variety of personal reasons for their visit preferences, which may include travel time, availability of transportation, mental health concerns, lack of support, reluctance or embarrassment about pelvic exams, a desire for reassurance from a surgeon, or any number of other rationales. In our experience, patients who feel strongly about the type of postoperative visit they prefer will make it clear to their surgeon and those patients should be accommodated, even in a practice that defaults to a different type of postoperative visit. In this study, two patients from each group switched to the other type of postoperative visit prior to their appointment, suggesting that patients have reasons that may drive them toward either visit type.

Strengths of this study include its randomized controlled design, the racially and socioeconomically diverse patient population, and the range of approaches and indications for hysterectomy, including pelvic organ prolapse, abnormal uterine bleeding, fibroids, and gender incongruence. Another strength is the survey questions utilized, which were targeted specifically to patient experience with postoperative visits and written at a reading level accessible to patients with lower health literacy. Limitations include recruitment from a single academic medical center, the exclusion of non-English-speaking patients, the use of non-validated survey questions, and the fact that only one quarter of participants were aged 50 or older, which may limit the generalizability of the findings to older patients.

Future research in this area might explore the cost effectiveness of telehealth, methods of improving telehealth for non-English speaking patients, and further inquiry about postoperative patient communication. Interesting research from the Netherlands has demonstrated the value of eHealth programs in reducing pain and time to return to work in patients who underwent gynecologic surgery [20]. These promising results may herald a future where eHealth applications are used to facilitate personalized postoperative communication with patients without clinic staff involvement. Additionally, qualitative studies could provide valuable insights into patients’ perceptions and experiences of telehealth in the postoperative setting, informing the development of tailored interventions to optimize patient-centered care delivery. Finally, it would be prudent to develop a validated postoperative satisfaction questionnaire given the inadequacy of available validated tools to assess patient experience with postoperative care.

In conclusion, this study demonstrates that postoperative telehealth visits are a viable alternative to clinic visits for appropriate patients. Our findings contribute to a growing body of evidence on the role of telehealth in gynecologic surgery and provide valuable insights into patient experience with telehealth visits. Embracing telehealth in surgical care delivery has the potential to enhance patient satisfaction, improve healthcare accessibility, and optimize resource utilization in the postoperative period.

## Supplementary Information

Below is the link to the electronic supplementary material.

Supplementary file1 (PDF 169 KB)

## Data Availability

The data that support the findings of this study are available on request from the corresponding author, SDW.

## Declarations

### Conference Presentation

American Urogynecologic Society PFD Week, Washington DC, October 24, 2024.
