# Supplementary material for: Telehealth vs Clinic Postoperative Visit After Hysterectomy: A Randomized Controlled Trial
Source: Int Urogynecol J. 2025 Jan 31;36(6):1229–36. doi: 10.1007/s00192-025-06070-9 (PMC12287227; doi:10.1007/s00192-025-06070-9)
Supplement: Supplementary file 1 — Supplementary file1 (PDF 169 KB) [file 192_2025_6070_MOESM1_ESM.pdf]

REDCap

Telehealth vs Clinic Post Op Visit after Minimally Invasive Hysterectomy
Record ID 92

Post-op visit survey

Record ID 92

My post-operative visit was...
* must provide value

- (•) in the office
- ( ) via telehealth

My surgeon listened carefully to me
* must provide value

Strongly disagree | Neutral | Strongly agree
Change the slider above to set a response
reset

My surgeon explained the surgical findings in a way that was easy to understand
* must provide value

Strongly disagree | Neutral | Strongly agree
Change the slider above to set a response
reset

My surgeon spent enough time with me
* must provide value

Strongly disagree | Neutral | Strongly agree
Change the slider above to set a response
reset

My questions regarding my surgery and recovery were answered
* must provide value

Strongly disagree | Neutral | Strongly agree
Change the slider above to set a response
reset

I achieved my treatment goals today
* must provide value

Strongly disagree | Neutral | Strongly agree
Change the slider above to set a response
reset

I would have preferred to have my postoperative appointment via telehealth
* must provide value

Strongly disagree | Neutral | Strongly agree
Change the slider above to set a response
reset

Overall, I was satisfied with my visit today
* must provide value

Strongly disagree — Neutral — Strongly agree

Change the slider above to set a response

reset

Have you previously experienced a Telehealth visit?
* must provide value

◯ Yes ◯ No

How long does it normally take you to travel to your surgeon's office (round trip)?
* must provide value

◯ Less than 15 minutes ◯ Between 15 and 30 minutes ◯ Between 30 and 60 minutes ◯ More than 60 minutes

How do you typically travel to your surgeon's office?
* must provide value

◯ Personal vehicle ◯ Ride share/Taxi/Car service ◯ Public Transportation ◯ Walk ◯ Friend or Family member's vehicle

Form Status

Complete?

Incomplete

REDCap

Telehealth vs Clinic Post Op Visit after Minimally Invasive Hysterectomy
Record ID 92

Post-op visit survey

Record ID 92

My post-operative visit was...
* must provide value

- ( ) in the office
- (•) via telehealth

My surgeon listened carefully to me
* must provide value

Strongly disagree | Neutral | Strongly agree

Change the slider above to set a response

reset

My surgeon explained the surgical findings in a way that was easy to understand
* must provide value

Strongly disagree | Neutral | Strongly agree

Change the slider above to set a response

reset

My surgeon spent enough time with me
* must provide value

Strongly disagree | Neutral | Strongly agree

Change the slider above to set a response

reset

My questions regarding my surgery and recovery were answered
* must provide value

Strongly disagree | Neutral | Strongly agree

Change the slider above to set a response

reset

I achieved my treatment goals today
* must provide value

Strongly disagree | Neutral | Strongly agree

Change the slider above to set a response

reset

Telehealth made it convenient for me to have an appointment with my surgeon
* must provide value

Strongly disagree | Neutral | Strongly agree

Change the slider above to set a response

reset

I would have preferred to have my postoperative appointment in person

* must provide value

Strongly disagree | Neutral | Strongly agree

Change the slider above to set a response

reset

I would use telehealth again

* must provide value

Strongly disagree | Neutral | Strongly agree

Change the slider above to set a response

reset

I would recommend telehealth to someone in my position

* must provide value

Strongly disagree | Neutral | Strongly agree

Change the slider above to set a response

reset

Overall, I was satisfied with my visit today

* must provide value

Strongly disagree | Neutral | Strongly agree

Change the slider above to set a response

reset

Have you previously experienced a Telehealth visit?

* must provide value

○ Yes ○ No

How long does it normally take you to travel to your surgeon's office (round trip)?

* must provide value

○ Less than 15 minutes ○ Between 15 and 30 minutes ○ Between 30 and 60 minutes ○ More than 60 minutes

How do you typically travel to your surgeon's office?

* must provide value

○ Personal vehicle ○ Ride share/Taxi/Car service ○ Public Transportation ○ Walk ○ Friend or Family member's vehicle

Form Status

Complete?

Incomplete
